# Long non-coding RNA CERS6-AS1 facilitates the oncogenicity of pancreatic ductal adenocarcinoma by regulating the microRNA-15a-5p/FGFR1 axis

**DOI:** 10.18632/aging.202540

**Published:** 2021-02-13

**Authors:** Zhennan Yun, Fanqi Meng, Shiquan Li, Ping Zhang

**Affiliations:** 1 Department of Colorectal and Anal Surgery, The First Hospital of Jilin University, Changchun 130021, Jilin, China; 2 Department of Hepatobiliary and Pancreatic Surgery, The First Hospital of Jilin University, Changchun 130021, Jilin, China

**Keywords:** CERS6 antisense RNA 1, ceRNA theory, pancreatic ductal adenocarcinoma, microRNA-15a-5p, fibroblast growth factor receptor 1

## Abstract

The long non-coding RNA CERS6 antisense RNA 1 (CERS6-AS1) has critical regulatory roles in breast cancer progression. Here, we determined CERS6-AS1 expression in pancreatic ductal adenocarcinoma (PDAC) and the roles of CERS6-AS1 in PDAC carcinogenesis. The mechanisms underlying the regulatory actions of CERS6-AS1 in PDAC cells were elucidated in detail. CERS6-AS1 expression was evidently increased in PDAC tissues and cell lines. Patients with PDAC having high CERS6-AS1 expression had shorter overall survival periods than those having low CERS6-AS1 expression. Functionally, the knockdown of CERS6-AS1 attenuated the proliferation, migration, and invasion and stimulated apoptosis of PDAC cells *in vitro*. Additionally, CERS6-AS1 depletion decreased PDAC tumor growth *in vivo.* Mechanistically, CERS6-AS1 could competitively bind to microRNA-15a-5p (miR-15a-5p) and effectively work as a molecular sponge in PDAC cells, resulting in the upregulation of fibroblast growth factor receptor 1 (FGFR1), a direct target of miR-15a-5p. Rescue experiments revealed that miR-15a-5p downregulation or FGFR1 restoration rescued the effects of CERS6-AS1 knockdown on the behaviors of PDAC cells. In conclusion, CERS6-AS1 promoted the oncogenicity of PDAC by serving as a competing endogenous RNA to sequester miR-15a-5p and increase FGFR1 expression, which highlights the potential of the CERS6-AS1/miR-15a-5p/FGFR1 pathway as an effective target for cancer therapy.

## INTRODUCTION

Pancreatic cancer significantly contributes to cancer-associated mortality worldwide, and it is characterized by aggressive local invasion and high metastatic potential [1]. Approximately 80% of pancreatic cancer cases are of the pancreatic ductal adenocarcinoma (PDAC) arising from pancreatic ductal epithelial cells [2]. Curative surgical excision is currently the most effective therapy for PDAC; however, approximately 80% of patients with PDAC are not candidates for this surgery owing to a delayed diagnosis, which prevents the management of the disease at an optimal time [3]. Despite continuous improvements in diagnostic methods and treatment strategies, the clinical outcomes of patients with PDAC remain poor [4]. In this regard, comprehensively elucidating the molecular events underlying tumor pathogenesis is vital for developing novel diagnostic and treatment strategies in PDAC.

Long non-coding RNAs (lncRNAs) are non-coding transcripts comprising >200 nucleotides [5]. They have almost no protein-coding abilities but are capable of regulating cellular behaviors, including epigenetic regulation, alternative splicing, RNA decay, cell growth, and differentiation [6]. Previous studies highlighted the involvement of lncRNAs in carcinogenesis and cancer progression [7–9]. In recent years, multiple lncRNAs are found to be differentially expressed in PDAC and perform critical roles in regulating the oncogenesis and progression of PDAC [10–12]. In addition, lncRNAs may exhibit cancer-promoting or cancer-inhibiting effects and are implicated in the control of malignant properties of carcinogenesis [13, 14]. For instance, PSMB8-AS1 [15], PCAT6 [16], and LINC00514 [17] are overexpressed in PDAC, and promote the aggressive properties. In contrast, LINC00261 [18], PXN-AS1 [19] and CASC2 [20] are weakly expressed, and inhibit the malignancy of PDAC.

MicroRNAs (miRNAs) represent one subgroup of small non-coding RNA molecules comprising 19–25 nucleotides [21]. miRNAs have emerged as regulatory transcripts and can negatively regulate genes expression [22]. Regarding the mechanism, a competing endogenous RNA (ceRNA) theory has been proposed that describes the crosstalk among lncRNAs, miRNAs, and mRNAs [23, 24]. LncRNAs can adsorb certain miRNAs and prevent the inhibition of mRNAs targeted by miRNAs, thereby regulating the oncogenicity of cancer [25]. Thus, studying the lncRNAs and miRNAs that contribute to PDAC tumorigenesis may provide valuable information for the development of anticancer treatment options.

The lncRNA CERS6 antisense RNA 1 (CERS6-AS1) plays critical regulatory roles in breast cancer progression [26]. Yet, the involvement of CERS6-AS1 in PDAC oncogenicity has not been characterized. Herein, we attempted to measure CERS6-AS1 expression in PDAC and further explore the functions of CERS6-AS1 in PDAC carcinogenesis. The molecular events responsible for the regulatory roles of CERS6-AS1 in PDAC were elucidated too. The identified CERS6-AS1/miR-15a-5p/FGFR1 pathway may offer a foundation for the identification of alternative therapies for PDAC.

## RESULTS

### The siRNA-mediated knockdown of CERS6-AS1 exerts tumor-suppressing effects on PDAC cells

**Figure 1:**
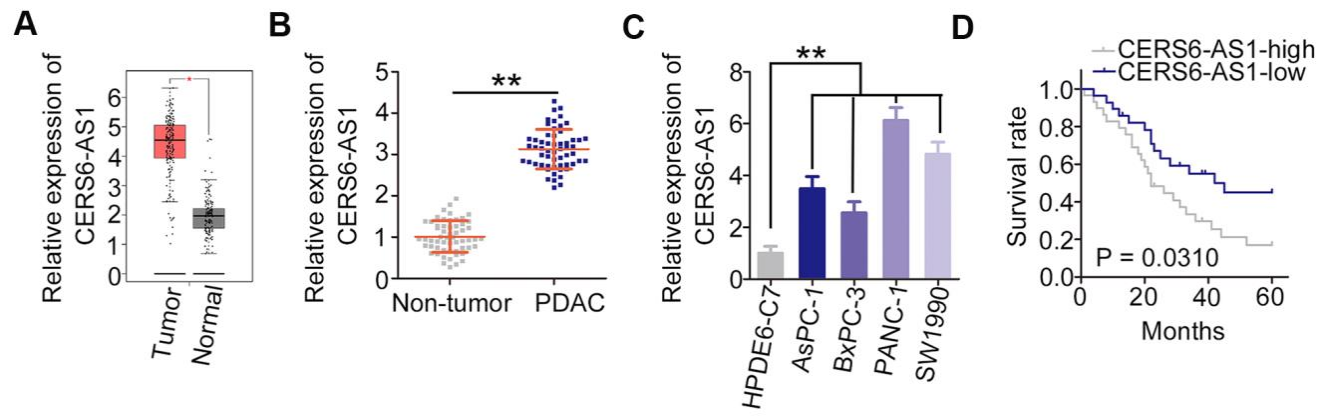
**CERS6-AS1 is upregulated in PDAC tissues and cell lines.** (**A**) GEPIA analysis of CERS6-AS1 expression in PDAC tissues. (**B**) CERS6-AS1 expression in PDAC tissues and adjacent non-tumor tissues was detected via RT-qPCR. (**C**) Relative expression of CERS6-AS1 in four PDAC cell lines was determined by RT-qPCR. (**D**) The correlation between CERS6-AS1 expression and overall survival in patients with PDAC was analyzed by the Kaplan–Meier method. **P < 0.01.

First, the expression profile of lncRNAs in PDAC was assessed employing Gene Expression Profiling Interactive Analysis (http://gepia.cancer-pku.cn/). Of these lncRNAs, CERS6-AS1 was one of the most notably highly expressed lncRNAs (Figure 1A). Consistently, CERS6-AS1 expression was higher in PDAC tissues relative to the adjacent non-tumor tissues (Figure 1B). Additionally, the overexpressed CERS6-AS1 was also validated in all four tested PDAC cell lines (Figure 1C). We then categorized PDAC patients into two groups based on median CERS6-AS1 expression within this patient cohort, and found that CERS6-AS1-high patients exhibited a significantly shorter overall survival relative to CERS6-AS1-low patients (Figure 1D; P = 0.0310).

**Figure 2:**
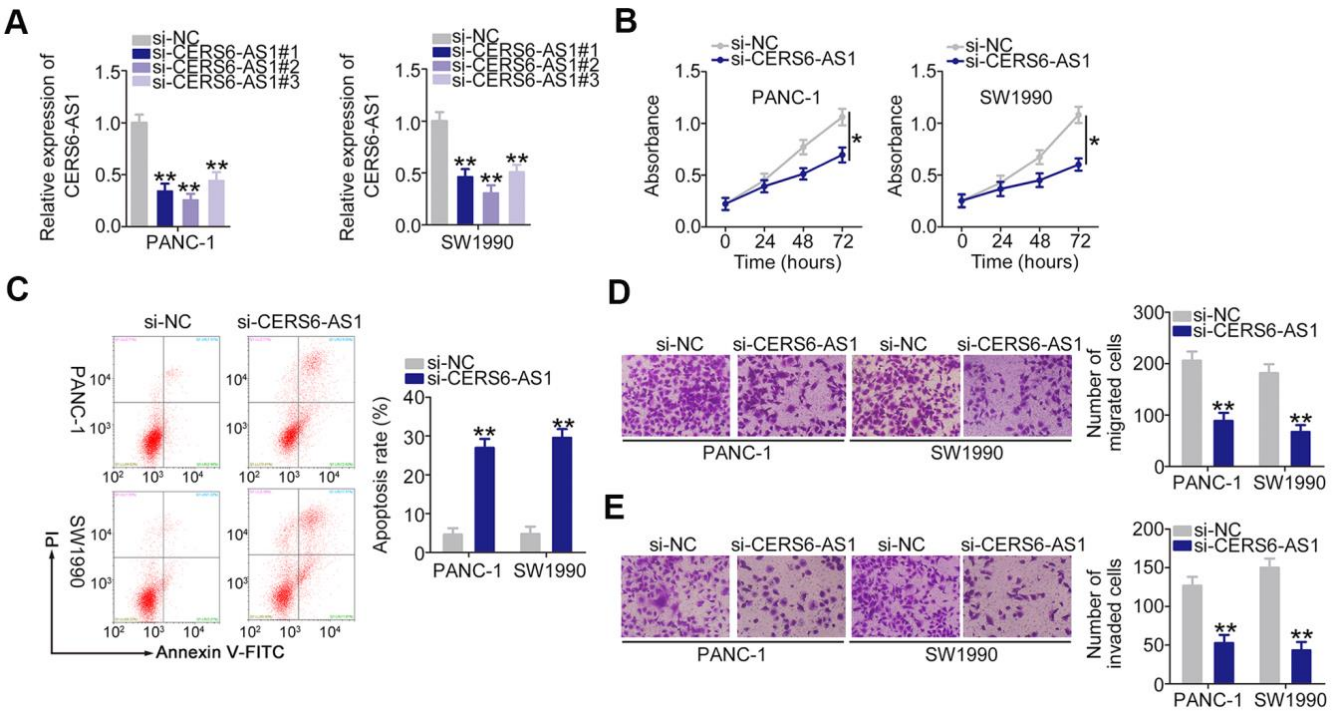
**Downregulation of CERS6-AS1 suppresses PDAC cell proliferation, migration, and invasion and induces cell apoptosis *in vitro*.** (**A**) CERS6-AS1 expression was detected by RT-qPCR in PANC-1 and SW1990 cells after treatment with si-CERS6-AS1 or si-NC. (**B**) CCK-8 assays were performed to measure the proliferation of CERS6-AS1-depleted PANC-1 and SW1990 cells. (**C**) Flow cytometry analysis was used to measure the apoptosis of PANC-1 and SW1990 cells after transfection with si-CERS6-AS1 or si-NC. (**D**, **E**) The migration and invasion in PANC-1 and SW1990 cells after CERS6-AS1 silencing was determined with Transwell cell migration and invasion assays. *P < 0.05 and **P < 0.01.

To explore whether CERS6-AS1 is functionally implicated in PDAC progression, specific siRNAs targeting CERS6-AS1 were employed to knock down endogenous CERS6-AS1 expression. All three siRNAs decreased the expression of CERS6-AS1 in PANC-1 and SW1990 cells (Figure 2A). si-CERS6-AS1#2 presented the highest silencing efficiency and was selected for loss-of-function assays. CERS6-AS1 silencing obviously lowered the proliferative capacity of PANC-1 and SW1990 cells (Figure 2B). Flow cytometry analysis manifested that the apoptosis rate evidently increased in both PDAC cell lines after CERS6-AS1 depletion (Figure 2C). Additionally, the migratory (Figure 2D) and invasive (Figure 2E) abilities of PANC-1 and SW1990 cells were weakened after CERS6-AS1 knockdown. Cumulatively, CERS6-AS1 may perform oncogenic roles in PDAC.

### CERS6-AS1 executes as an miR-15a-5p sponge in PDAC

**Figure 3:**
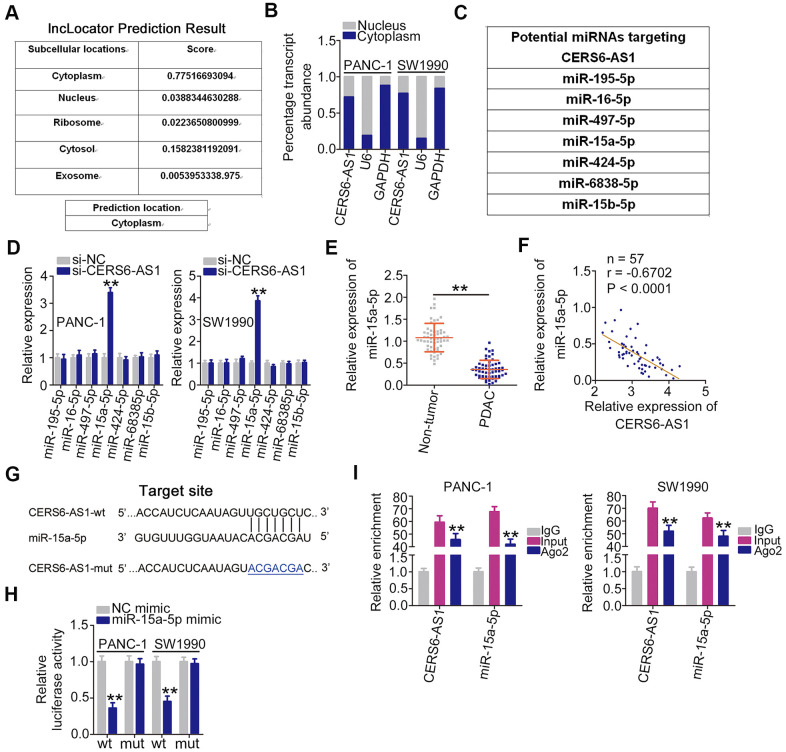
**CERS6-AS1 sequesters miR-15a-5p in PDAC cells.** (**A**) The location of CERS6-AS1 predicted by lncLocator. (**B**) CERS6-AS1 distribution in the cytoplasmic and nuclear fractions of PANC-1 and SW1990 cells. (**C**) The potential miRNAs of CERS6-AS1 predicted by StarBase 2.0. (**D**) Relative miRNA expression was detected in PANC-1 and SW1990 cells after CERS6-AS1 knockdown. (**E**) miR-15a-5p expression in paired PDAC tissues and adjacent non-tumor tissues was detected by RT-qPCR. (**F**) The correlation between miR-15a-5p and CERS6-AS1 expression in PDAC tissues was examined by Pearson’s correlation coefficient analysis. (**G**) The wild-type and mutant miR-15a-5p binding sites in CERS6-AS1. (**H**) PANC-1 and SW1990 cells were transfected with CERS6-AS1-wt or CERS6-AS1-mut reporter vectors and miR-15a-5p mimic or NC mimic, and a luciferase reporter assay was conducted to measure the luciferase activity. (**I**) PANC-1 and SW1990 cells were incubated with Ago2 or IgG, and then immunoprecipitated RNA was analyzed by RT-qPCR to evaluate miR-15a-5p and CERS6-AS1 enrichment. **P < 0.01.

To elucidate the molecular events through which CERS6-AS1 executes its roles, lncLocator (http://www.csbio.sjtu.edu.cn/bioinf/lncLocator/) was employed to predict the distribution of CERS6-AS1. CERS6-AS1 is predicted to be mostly located in the cytoplasm (Figure 3A), which was further corroborate by cell cytoplasmic/nuclear fractionation assay (Figure 3B). The putative miRNAs with complementary sequences paring with CERS6-AS1 were predicted using the bioinformatics tool StarBase 2.0. As shown in Figure 3C, 7 miRNAs were predicted to harbor potential target sites for CERS6-AS1. Then, the expression of these candidates was determined in CERS6-AS1-depleted PDAC cells. miR-15a-5p was strikingly increased after CERS6-AS1 silencing, whereas there was no change in the expression of other candidates (Figure 3D). Additionally, miR-15a-5p was weakly expressed in PDAC tissues (Figure 3E), which was inversely correlated with CERS6-AS1 expression (Figure 3F).

The luciferase reporter assay was performed to determine whether CERS6-AS1 directly binds to miR-15a-5p in PDAC cells (Figure 3G). In PANC-1 and SW1990 cells, exogenous miR-15a-5p expression clearly lowered the luciferase activity of CERS6-AS1-wt in PANC-1 and SW1990 cells, whereas the luciferase activity of CERS6-AS1-mut was unaltered after miR-15a-5p upregulation (Figure 3H). Furthermore, CERS6-AS1 and miR-15a-5p were co-immunoprecipitated by the anti-Ago2 antibody in PANC-1 and SW1990 cells (Figure 3I). Overall, CERS6-AS1 acted as a ceRNA and directly sponged miR-15a-5p in PDAC.

### FGFR1 is a direct target of miR-15a-5p in PDAC cells

**Figure 4:**
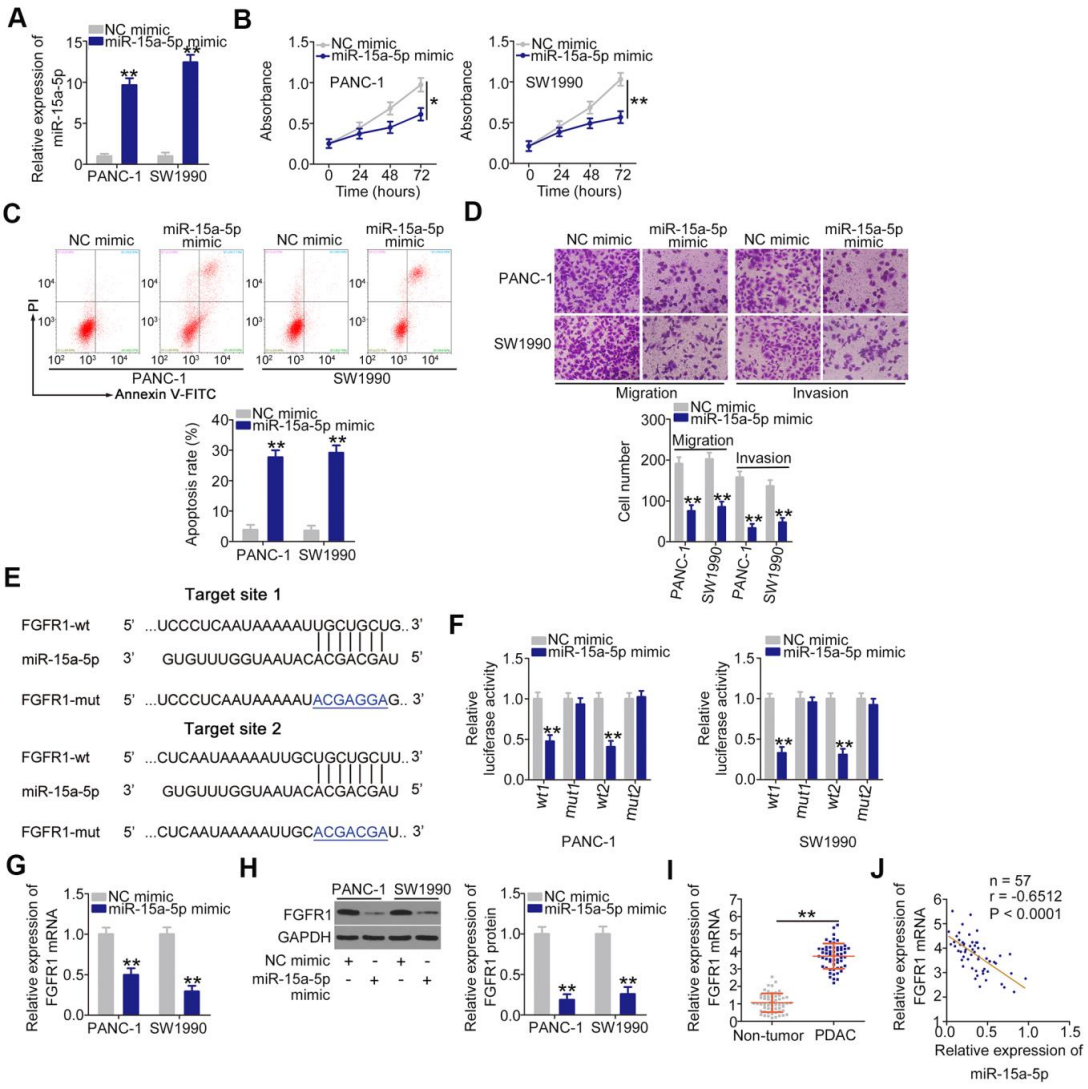
**miR-15a-5p plays anti-oncogenic roles and directly targets FGFR1 in PDAC.** (**A**) miR-15a-5p mimic or NC mimic was introduced into PANC-1 and SW1990 cells, and miR-15a-5p expression was measured by RT-qPCR. (**B**, **C**) The proliferation and apoptosis of PANC-1 and SW1990 cells following miR-15a-5p overexpression were measured by CCK-8 assays and flow cytometry analysis, respectively. (**D**) Transwell cell migration and invasion assays were performed to assess the migration and invasion of PANC-1 and SW1990 cells after miR-15a-5p mimic or NC mimic transfection. (**E**) The miR-15a-5p binding sites in FGFR1. (**F**) Luciferase activity was detected in PANC-1 and SW1990 cells cotransfected with miR-15a-5p mimic or NC mimic and FGFR1-wt or FGFR1-mut. (**G**, **H**) RT-qPCR and western blotting were used to detect FGFR1 mRNA and protein expression in PANC-1 and SW1990 cells after miR-15a-5p overexpression. (**I**) RT-qPCR confirmation of FGFR1 mRNA expression in paired PDAC tissues and adjacent non-tumor tissues. (**J**) Pearson’s correlation coefficient was determined to analyze the correlation between FGFR1 mRNA and miR-15a-5p in PDAC tissues. *P < 0.05 and **P < 0.01.

The roles of miR-15a-5p overexpression on PDAC cells were then evaluated. The increase of miR-15a-5p in PDAC cells was achieved by transfecting with miR-15a-5p mimic (Figure 4A). Ectopic miR-15a-5p expression inhibited PANC-1 and SW1990 cell proliferation (Figure 4B), facilitate cell apoptosis (Figure 4C). In addition, transfection with miR-15a-5p mimic caused an obvious impairment in cell migration (Figure 4D) and invasion (Figure 4D) in PANC-1 and SW1990 cells. We subsequently identified the downstream target of miR-15a-5p in PDAC cells. Two miR-15a-5p binding sites were observed within the 3'-UTR region of FGFR1 (Figure 4E). The gene was chosen for further experimental confirmation owing to its well-known contribution to the oncogenesis and progression of PDAC [27, 28]. Luciferase reporter assay confirmed that miR-15a-5p mimic lowered the luciferase activity of FGFR1-wt (1 and 2; Figure 4F); nevertheless, this suppressive action was abrogated when the binding sites were mutated. Furthermore, the upregulation of miR-15a-5p reduced FGFR1 expression (Figure 4G, 4H) in PANC-1 and SW1990 cells. In addition, the level of FGFR1 mRNA was higher in PDAC tissues, and presented an inverse association with miR-15a-5p level (Figure 4J). Overall, these results identified FGFR1 as a direct miR-15a-5p target in PDAC.

### CERS6-AS1 works as a ceRNA for miR-15a-5p and thereby regulates the expression of FGFR1 PDAC cells

**Figure 5:**
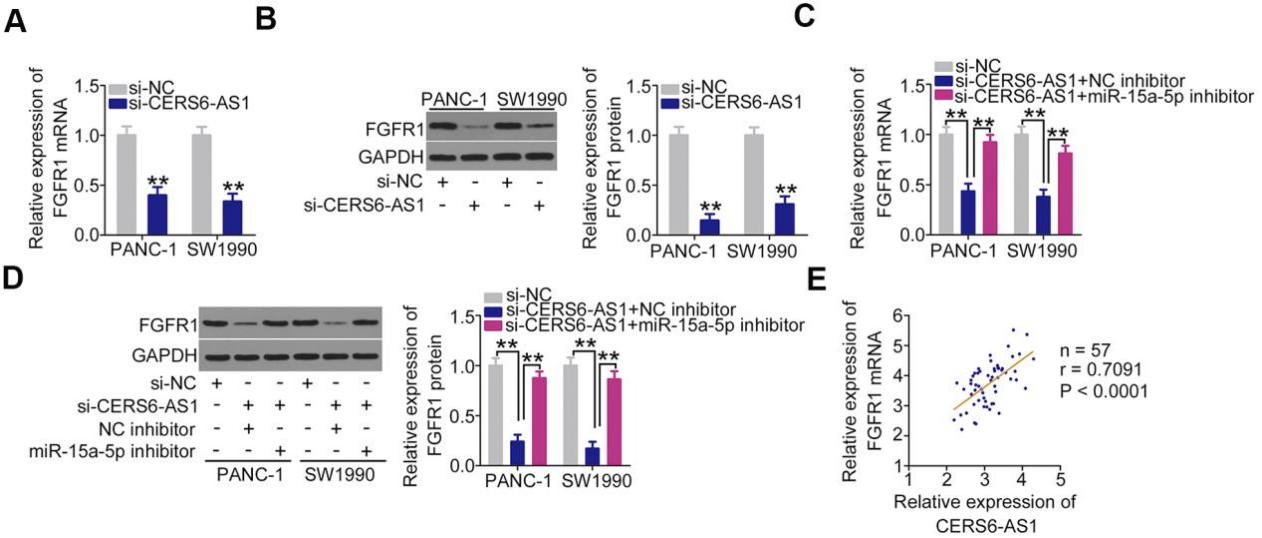
**CERS6-AS1 regulates FGFR1 expression in PDAC cells by sponging miR-15a-5p.** (**A**, **B**) CERS6-AS1-depleted PANC-1 and SW1990 cells were analyzed by RT-qPCR and western blotting to quantify FGFR1 mRNA and protein levels, respectively. (**C**, **D**) si-CERS6-AS1 was cotransfected with miR-15a-5p inhibitor or NC inhibitor into PANC-1 and SW1990 cells. After transfection, RT-qPCR and western blotting were used to determine the mRNA and protein levels of FGFR1. (**E**) The correlation between FGFR1 mRNA and CERS6-AS1 expression in PDAC tissues was determined by Pearson’s correlation coefficient analysis. **P < 0.01.

After confirming that CERS6-AS1 functions as an miR-15a-5p sponge, we next attempted to address investigate whether CERS6-AS1 controlled the expression of FGFR1 in PDAC cells. Loss of CERS6-AS1 caused a significant decrease in FGFR1 expression (Figure 5A, 5B) levels in PANC-1 and SW1990 cells. Interestingly, inhibiting miR-15a-5p abolished the inhibitory actions of CERS6-AS1 knockdown on FGFR1 expression (Figure 5C, 5D) in PANC-1 and SW1990 cells. Furthermore, a positive expression relationship between FGFR1 mRNA and CERS6-AS1 was confirmed in PDAC tissues (Figure 5E). Cumulatively, CERS6-AS1 positively regulated the expression of FGFR1 in PDAC cells via sequestering miR-15a-5p.

### CERS6-AS1 facilitates the aggressiveness of PDAC cells by controlling the miR-15a-5p/FGFR1 axis

**Figure 6:**
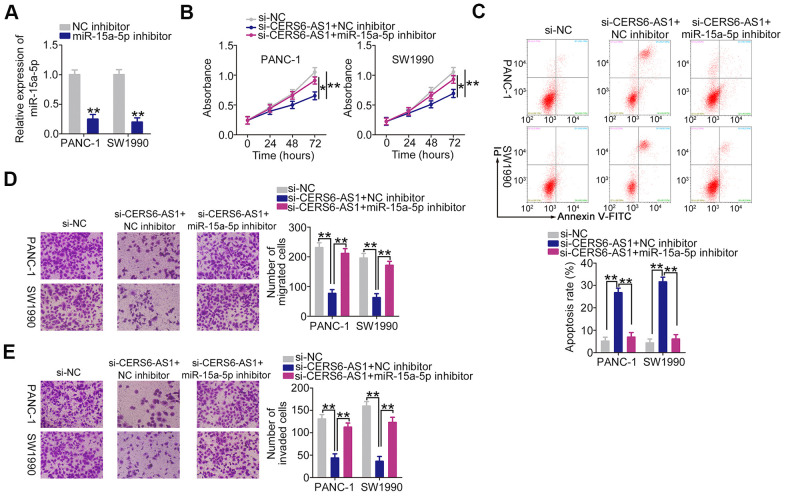
**miR-15a-5p inhibition abolishes the anti-oncogenic actions of CERS6-AS1 knockdown in PDAC cells.** (**A**) RT-qPCR analysis confirmation of miR-15a-5p expression in PANC-1 and SW1990 cells treated with miR-15a-5p inhibitor or NC inhibitor. (**B**, **C**) miR-15a-5p inhibitor or NC inhibitor, together with si-CERS6-AS1, was cotransfected into PANC-1 and SW1990 cells. Cell proliferation and apoptosis were detected by CCK-8 assays and flow cytometry analysis, respectively. (**D**, **E**) The migration and invasion of the cells described above were measured by Transwell cell migration and invasion assays. *P < 0.05 and **P < 0.01.

The contribution of miR-15a-5p/FGFR1 to the tumor-promoting effects of CERS6-AS1 in PDAC cells was assessed employing rescue experiments. The transfection efficiency of miR-15a-5p inhibitor is demonstrated in Figure 6A. PANC-1 and SW1990 cells were transfected with si-CERS6-AS1 and miR-15a-5p inhibitor either alone or in combination. Downregulation of CERS6-AS1 attenuated cell proliferation (Figure 6B) and facilitated cell apoptosis (Figure 6C); however, cotransfection with miR-15a-5p inhibitor reversed both of these effects. Furthermore, miR-15a-5p inhibition abolished the suppressive impacts of si-CERS6-AS1 on the migration (Figure 6D) and invasion (Figure 6E) of PANC-1 and SW1990 cells.

**Figure 7:**
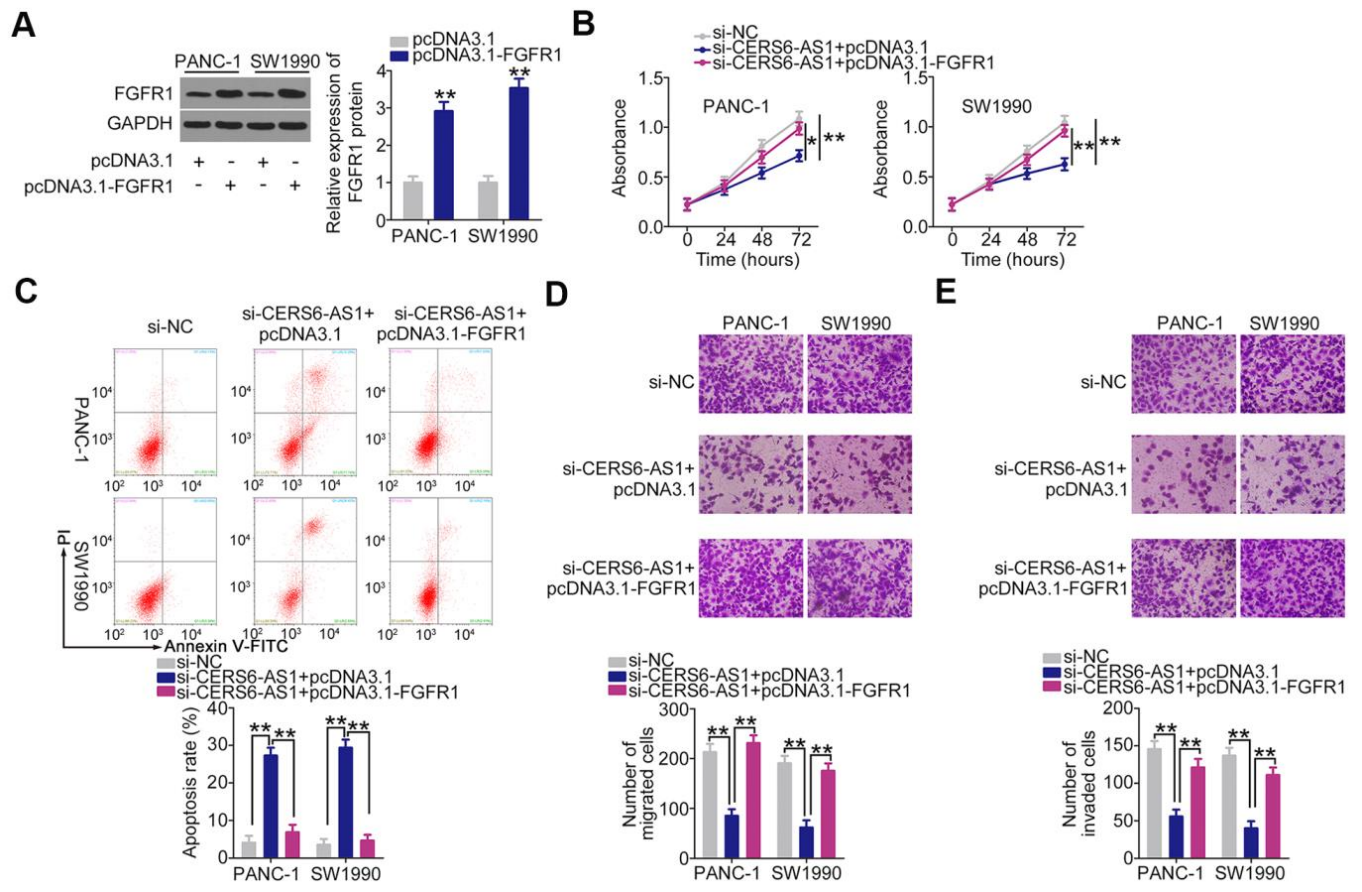
**FGFR1 overexpression counteracts the inhibitory effects of CERS6-AS1 silencing in PDAC cells.** (**A**) Western blot analysis was conducted to detect the expression of FGFR1 protein in PANC-1 and SW1990 cells after pcDNA3.1-FGFR1 or pcDNA3.1 transfection. (**B**–**E**) miR-15a-5p inhibitor or NC inhibitor was transfected into CERS6-AS1-depleted PANC-1 and SW1990 cells. The transfected cells were subjected to CCK-8 assay, flow cytometry analysis, and Transwell cell migration and invasion assays to analyze cell proliferation, apoptosis, and migration and invasion, respectively. *P < 0.05 and **P < 0.01.

For rescue experiments, the FGFR1 overexpression plasmid pcDNA3.1-FGFR1 was used to increase FGFR1 protein expression in PDAC cells (Figure 7A). The pcDNA3.1-FGFR1 or pcDNA3.1, together with si-CERS6-AS1, was introduced into PANC-1 and SW1990 cells. CERS6-AS1 upregulation effectively reversed the effects of si-CERS6-AS1 on the proliferation (Figure 7B), apoptosis (Figure 7C), migration (Figure 7D), and invasion (Figure 7E) of PANC-1 and SW1990 cells. Overall, CERS6-AS1 promotes the oncogenicity of PDAC cells by controlling miR-15a-5p/FGFR1 axis.

### CERS6-AS1 depletion suppresses the PDAC tumor growth *in vivo*

**Figure 8:**
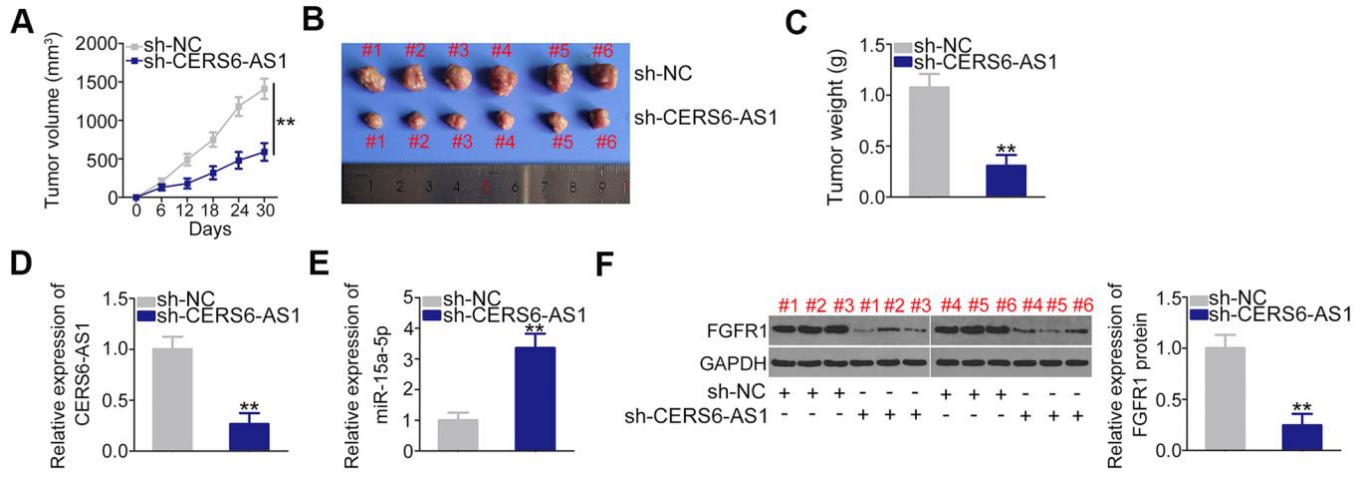
**CERS6-AS1 knockdown impairs the tumor growth of PDAC cells *in vivo*.** (**A**) Tumor growth was monitored by measuring tumor volumes in 6-day intervals. (**B**) Representative images of tumor xenografts obtained from the sh-CERS6-AS1 and sh-NC groups. (**C**) The tumor xenografts removed from mice at 30 days after the injection of SW1990 cells stably overexpressing sh-CERS6-AS1 or sh-NC. The weights of xenografts were detected. (**D**) The level of CERS6-AS1 in xenografts was evaluated by RT-qPCR. (**E**) miR-15a-5p expression in the xenografts was determined by RT-qPCR. (**F**) Western blotting was used to measure FGFR1 protein expression in xenografts. **P < 0.01.

Subcutaneous xenograft models were executed by injecting mice with SW1990 cells stably overexpressing sh-CERS6-AS1 or sh-NC. The results revealed that tumor growth (Figure 8A, 8B) and weight (Figure 8C) in the sh-CERS6-AS1 group was clearly inhibited in contrast to sh-NC group (Figure 8A, 8B). Furthermore, RT-qPCR analysis showed that the CERS6-AS1 was downregulated in the tumors originating from SW1990 cells stably expressing sh-CERS6-AS1 (Figure 8D). Moreover, the miR-15a-5p level was increased (Figure 8E) and FGFR1 protein was downregulated (Figure 8F) in the CERS6-AS1-silenced tumor xenografts. Therefore, these findings verified that CERS6-AS1 knockdown impaired PDAC tumor growth *in vivo*.

## DISCUSSION

Several studies have revealed that lncRNAs exhibit crucial functions in controlling complex cellular behaviors, and this has attracted considerable interest [29–31]. To date, a large number of lncRNAs have been validated as critical regulators of PDAC malignancy [32, 33]. Therefore, elucidating the detailed mechanisms via which lncRNAs contribute to the genesis and progression of PDAC might be advantageous for the development of effective targets for cancer management. Herein, we assessed the expression and functional importance of the lncRNA CERS6-AS in PDAC cells. Our results identified the oncogenic CERS6-AS1/miR-15a-5p/FGFR1 pathway in PDAC.

CERS6-AS1 expression is overexpressed in breast cancer [26], presenting a negative correlation with the overall survival [26]. Functionally, CERS6-AS1 upregulation induces breast cancer cell proliferation and colony-forming abilities and suppresses cell apoptosis *in vitro* [26]. However, whether CERS6-AS1 is implicated in PDAC requires further investigation. Here, a high CERS6-AS1 was validated in PDAC. PDAC patients expressing high levels of CERS6-AS1 exhibited shorter overall survival relative to patients expressing low levels of this lncRNA. Functional analysis demonstrated that CERS6-AS1 knockdown restricted the *in vitro* properties of proliferative, migratory, and invasive, facilitated cell apoptosis *in vitro*, and impaired tumor growth *in vivo* of PDAC cells. Accordingly, CERS6-AS1 worked as a cancer-facilitating lncRNA in PDAC, and it may be an effective target for PDAC diagnosis and management.

Regarding the mechanism of action of lncRNAs, growing research has confirmed the existence of extensive ceRNA networks [25]. lncRNAs can sequester certain miRNAs and prevent their binding to target mRNAs, thereby weakening the inhibitory effect of miRNAs on their target genes [34]. To determine whether CERS6-AS1 affects PDAC malignancy through this mechanism, its location in PDAC cells was first analyzed by lncLocator and cell cytoplasmic/nuclear fractionation assays. CERS6-AS1 was corroborated to be mostly distributed in PDAC cell cytoplasm. Hence, bioinformatics analysis was used to identify the potential miRNAs with complementarity to CERS6-AS1, and the prediction unveiled that miR-15a-5p interacts with CERS6-AS1. Next, CERS6-AS1 knockdown resulted in the overexpression of miR-15a-5p in PDAC cells. Additionally, miR-15a-5p was underexpressed in PDAC and presented a negative correlation with the CERS6-AS1 level. Further investigations, including luciferase reporter assay and RIP, verified the direct binding between CERS6-AS1 and miR-15a-5p in PDAC.

It is well established that lncRNAs have miRNA response elements and sequester miRNAs to positively modulate the downstream target mRNAs of miRNAs [35]. After identifying that miR-15a-5p directly targets FGFR1, we next examined whether CERS6-AS1 regulates the expression of FGFR1 by decoying miR-15a-5p in PDAC. To this end, FGFR1 expression in CERS6-AS1-deficient PDAC cells was detected, and the data uncovered that CERS6-AS1 silencing reduced FGFR1 amounts in PDAC cells, whereas miR-15a-5p inhibition partially reversed this effect. Importantly, FGFR1 expression was increased in PDAC and positively correlated with CERS6-AS1. Together, these results confirmed a new ceRNA model in PDAC cells, comprising of CERS6-AS1, miR-15a-5p, and FGFR1.

miR-15a-5p is weakly expressed in various human cancers, including PDAC [36]. Patients with PDAC manifesting low miR-15a-5p level have shorter overall survival relative to those patients with high miR-15a-5p expression [37]. Functionally, exogenous miR-15a-5p expression decreases cell growth, metastasis, and epithelial to mesenchymal transition as well as increases cell cycle arrest in PDAC [36–38]. Mechanistically, the link between miR-15a-5p and FGFR1 was first demonstrated in PDAC, and miR-15a-5p exerted its anti-oncogenic activities by directly targeting FGFR1. FGFR1, belongs to the fibroblast growth factor family [39], is highly expressed in PDAC [40]. FGFR1 executes cancer-promoting actions in PDAC oncogenesis [27, 28], and affects a number of biological behaviors [28, 41–43]. However, the upstream mechanisms that control FGFR1 expression remain unclear. In this study, our data validated that FGFR1 is regulated by CERS6-AS1 via its competitive binding to miR-15a-5p. Importantly, FGFR1 overexpression or miR-15a-5p downregulation abrogated the anti-cancer activities of CERS6-AS1 deficiency on the malignant characteristics of PDAC cells. Altogether, our study provided convincing evidence that FGFR1 is regulated by CERS6-AS1 via the sponging of miR-15a-5p, and the miR-15a-5p/FGFR1 axis mediates the actions of CERS6-AS1 in PDAC.

Our study performed luciferase reporter assay, RT-qPCR, western blotting, and relationship analysis and confirmed that miR-15a-5p directly targeted FGFR1 in PDAC. However, ChIP assay was not implemented to confirm this interaction, and it was a limitation of our study.

## CONCLUSIONS

**Figure 9:**
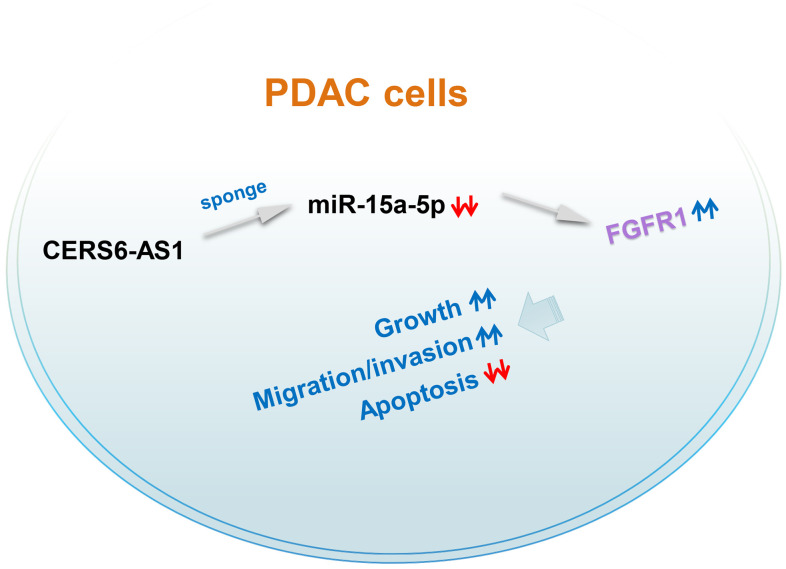
**Schematic diagram presents the CERS6-AS1/miR-15a-5p/FGFR1 pathway in PDAC.** CERS6-AS1 aggravates the malignancy of PDAC cells by exerting as an miR-15a-5p sponge and consequently increasing FGFR1 expression.

CERS6-AS1 was upregulated in PDAC, and CERS6-AS1 knockdown clearly suppressed PDAC progression. CERS6-AS1 acted as an miR-15a-5p sponge in PDAC cells and thus increased FGFR1 expression, thereby performing critical roles in the tumorigenesis of PDAC (Figure 9). Altogether, our data offer new insights into the mechanisms of PDAC pathogenesis, and suggest that the CERS6-AS1/miR-15a-5p/FGFR1 axis may represent a viable therapeutic target in PDAC therapy.

## MATERIALS AND METHODS

### Tissue specimen collection and cell culture

Human PDAC tissues and adjacent non-tumor tissues were acquired from 57 patients in The First Hospital of Jilin University. All patients had not received either local or systemic anticancer therapies before surgery. Approval was obtained from the Ethics Committee of The First Hospital of Jilin University (ECTFHJU.2015-0112). All patients signed informed consent at the initial stage of the study.

PDAC cell lines, including AsPC-1, BxPC-3, and PANC-1 and SW1990, were bought from the Cell Bank of the Chinese Academy of Sciences (Shanghai, China). SW1990 cells were grown in L-15 culture medium (Gibco, Grand Island, NY, USA) containing 10% fetal bovine serum (FBS; Gibco). RPMI-1640 medium (Gibco) added with 10% FBS was used in the culture of other three PDAC cell lines. HPDE6-C7 (BNCC338285; BeNa Culture Collection, Beijing, China), a normal human pancreatic cell line, was grown in DMEM (Gibco) containing with 10% FBS. In addition, all cell culture media contained 1% penicillin/streptomycin (Gibco). All cells were cultured at 37° C in an incubator equipped with 5% CO_2_.

### Cell transfection

RiboBio (Guangzhou, China) synthesized small interfering RNAs (siRNAs) specifically for CERS6-AS1 (si-CERS6-AS1) and corresponding negative control siRNA (si-NC). The FGFR1 overexpression plasmid pcDNA3.1-FGFR1, miR-15a-5p mimic, miRNA mimic negative control (NC mimic), miR-15a-5p inhibitor and negative control inhibitor (NC inhibitor) were all bought from GenePharma (Shanghai, China). All transient transfections were performed employing Lipofectamine 2000 (Invitrogen, Shanghai, China).

### RT-qPCR

Using TRIzol (Invitrogen; Thermo Fisher Scientific, Inc.), total RNA was extracted, after which the RNA quantity and quality was measured with a NanoPhotometer spectrometer (Thermo Fisher Scientific, Inc.). The synthesis of complementary DNA (cDNA) was executed using a PrimeScript™ RT Reagent Kit with gDNA Eraser (Takara, Dalian, China). Next, qPCR was performed to determine CERS6-AS1 and FGFR1 expression utilizing TB Green Premix Ex Taq (Takara). The internal control for CERS6-AS1 and FGFR1 expression was glycerol 3-phosphate dehydrogenase (GAPDH).

Small RNA was isolated from analyzed samples or cells using the RNAiso for Small RNA Kit (Takara). In order to determine miR-15a-5p expression, Mir-X miRNA First-Strand Synthesis Kit and Mir-X miRNA qRT-PCR TB Green® Kit (Takara) were used for, respectively, conducting reverse transcription and qPCR. U6 small nuclear RNA served as a normalization control. The data were analyzed using the 2^–ΔΔCt^ approach.

### Cell cytoplasmic/nuclear fractionation

In order to separate the nuclear and cytoplasmic fractions of PDAC cells, a Nuclear/Cytosol Fractionation Kit (Cell Biolabs, CA, USA) was utilized, after which RT-qPCR was done to test CERS6-AS1 distribution.

### CCK-8 assay

At 24 h post-transfection, 2 × 10^3^ cells in 100-μ;l aliquots of culture medium were seeded into each well of 96-well plates. At different times after cell seeding, 10 μl of the CCK-8 reagent (Dojido, Kumamoto, Japan) was introduced into each well, after which were cultivated for additional 2 h at 37° C. In the following step, a microplate reader (PerkinElmer, MA, USA) was utilized to read the absorbance at a 450 nm wavelength.

### Flow cytometry analysis

Annexin V- FITC Apoptosis Detection Kit (BioLegend, CA, USA) was employed to assess PDAC cell apoptosis. Briefly, transfected cells were collected by trypsin (0.25%) digestion and resuspended in 100 μl binding buffer. Next, these cells were double-stained using 5 μl each of Annexin V-FITC and PI. After 20 min cultivation in the dark, a FACSCalibur Flow Cytometer was used to analyze cell apoptosis.

### Transwell cell migration and invasion assays

To assess cell migration, cells were collected 48 h after transfection. Cells were then resuspended in FBS-free culture medium, and the cell concentration was adjusted to 5 × 10^5^ cells/ml. Thereafter, the upper portion of transwell chambers (8-μm pore inserts; Corning, NY, USA) was loaded with a 100-μl cell suspension. Simultaneously, 700 μl of culture medium added with 20% FBS was introduced into the lower chamber as a chemoattractant. The transwell chambers were then allowed for 24 h incubation, after which the migrated cells were fixed and stained, respectively, utilizing 4% paraformaldehyde and 0.05% crystal violet. After washing and drying, the migrated cells in five random fields of view were counted. Cell invasion was quantified using the same protocol, with transwell inserts having been coated with Matrigel (BD Biosciences) prior to cell inoculation.

### Xenograft assay

The Institutional Animal Care and Use Committee of The First Hospital of Jilin University (ACUCTFHJU.2015-0112) approved the present animal study. A lentivirus expressing a short hairpin RNA (shRNA) against CERS6-AS1 (sh-CERS6-AS1) and negative control shRNA (sh-NC) were designed and packaged by Genechem (Shanghai, China). SW1990 cells were injected with the lentivirus to generate a cell line with stable CERS6-AS1 knockdown. To establish a tumor xenograft model system, male BALB/c nude mice (4-week-old; Laboratory Animal Center of Shanghai Academy of Science, Shanghai, China) were subcutaneously injected with SW1990 cells (2 × 10^6^) stably expressing sh-CERS6-AS1 or sh-NC (n = 6 each group). Tumor width (W) and length (L) were monitored every 6 days for a total of 30 days, and tumor volumes were calculated using the formula V =  0.5 × (L × W^2^). In the end, all mice were euthanized, and tumors were collected, weighted, and imaged as appropriate.

### Bioinformatics analysis

StarBase 3.0 (http://starbase.sysu.edu.cn/) was adopted to identify the miRNAs targeting CERS6-AS1. The putative targets of miR-15a-5p were found with three online databases, namely TargetScan (http://www.targetscan.org/), miRDB (http://mirdb.org/), and StarBase 3.0.

### Luciferase reporter assay

The wild-type (wt) CERS6-AS1 and FGFR1 fragments containing putative miR-15a-5p binding site were prepared and cloned into the psiCHECK-2 reporter vectors (Promega, Madison, WI, USA) to yield the CERS6-AS1-wt and FGFR1-wt reporter constructs. The mutated (mut) versions of the fragments were additionally prepared with the GeneTailor™ Site-Directed Mutagenesis System (Invitrogen), after which CERS6-AS1-mut and FGFR1-mut reporter constructs were generated as above. After growing until 70%–80% confluence, cotransfection of wt or mut reporter vectors and miR-15a-5p mimic or NC mimic was implemented using Lipofectamine 2000. Forty-eight h later, the Dual-Luciferase Reporter Assay System (Promega) was utilized for luciferase activity determination.

### RIP assay

The possible interactions between miR-15a-5p and CERS6-AS1 were examined with a Magna RIP RNA-Binding Protein Immunoprecipitation Kit (Millipore, Bedford, MA, USA). RIP lysis buffer was utilized to lyse PDAC cells, after which 10 μl of the cell lysate was retained as input and 100 μl of the cell lysate were probed overnight at 4° C using magnetic beads conjugated with either normal mouse IgG or human anti-Ago2 antibody (Millipore) suspended in 900 μl of RIP lysis buffer. Magnetic beads were then collected and treated with Proteinase K to digest the proteins. The immunoprecipitated RNA was evaluated via RT-qPCR to measure CERS6-AS1 and miR-15a-5p enrichment.

### Western blot analysis

RIPA buffer containing protease and phosphatase inhibitor cocktails (Beyotime, Shanghai, China) was utilized to lyse cells. Then, protein concentrations were assessed with a Detergent Compatible Bradford Protein Assay Kit (Beyotime). Using 10% SDS-PAGE, the protein was equally separated and then transferred to PVDF membranes, followed by blocking for a period of 2 h at room temperature using 5% nonfat milk. Subsequently, the membranes were probed overnight with anti-FGFR1 (ab76464; Abcam, Cambridge, UK) and anti-GAPDH (ab128915; Abcam) primary antibodies at 4° C, rinsed with TBST thrice, and probed for 1 h with a secondary HRP- conjugated antibody (ab205718; Abcam). Protein was visualized with BeyoECL Plus (Abcam). GAPDH functioned as the endogenous reference.

### Statistical analysis

All measured data were exhibited as the mean ± SD. Statistical analysis was executed using SPSS software 22.0 (SPSS, Chicago, IL, USA). Data analysis between groups was done utilizing Student’s t-test and one-way analysis of variance. The correlations among CERS6-AS1, miR-15a-5p, and FGFR1 levels in PDAC tissues were tested via Pearson’s correlation coefficient. Using the median CERS6-AS1 value, all PDAC patients were stratified into either CERS6-AS1-low or CERS6-AS1-high groups, after which their survival analysis was conducted employing Kaplan–Meier method and the log-rank test. P < 0.05 indicated a statistical significance.
